# Patient Safety Incident Reporting and Learning Guidelines Implemented by Health Care Professionals in Specialized Care Units: Scoping Review

**DOI:** 10.2196/48580

**Published:** 2024-10-04

**Authors:** Tusiwe Mabel Gqaleni, Sipho Wellington Mkhize, Geldine Chironda

**Affiliations:** 1 School of Nursing and Public Health University of KwaZulu-Natal Durban South Africa

**Keywords:** patient safety incidents, adverse events, harm, near misses, reporting guidelines, implementation guidelines, implementation practices, intervention strategies, critical care units, intensive care units, patient safety, specialized care unit, guidelines, clinical practice, healthcare professional, ICU

## Abstract

**Background:**

Implementing Patient Safety Incident Reporting and Learning (PSIRL) guidelines is critical in guiding clinical practice and improving clinical outcomes in specialized care units (SCUs). There is limited research on the evidence of the implemented PSIRL guidelines in SCUs at the global level.

**Objective:**

This review aims to map the evidence of PSIRL guidelines implemented by health care professionals in specialized care units globally.

**Methods:**

A scoping review methodology, according to Joanna Briggs Institute, was adopted. The eligibility criteria were guided by the Population, Concept, and Context (PCC) framework, with the Population including health care professionals, the Concept including PSIRL guidelines, and the Context including specialized units globally. Papers written in English were searched from relevant databases and search engines. The PRISMA-ScR (Preferred Reporting Items for Scoping Reviews and Meta-Analyses extension for Scoping Reviews) checklist for used.

**Results:**

The 13 selected studies were published from 2003 to 2023. Most articles are from the Netherlands and Switzerland (n=3), followed by South Africa (n=2). The nature of implemented PSIRL guidelines was computer-based (n=11) and paper-based incident reporting (n=2). The reporting system was intended for all the health care professionals within the specialized units, focusing on patients, staff members, and families. The outcomes of implemented incident reporting guidelines were positive, as evidenced by improved reporting of incidents, including medication errors (n=8) and decreased rate of incidents and errors (n=4). Furthermore, 1 study showed no change (n=1) in implementing the incident reporting guidelines.

**Conclusions:**

The implementation of reporting of patient safety incidents (PSIs) in specialized units started to be reported around 2002; however, the frequency of yearly publications remains very low. Although some specialized units are still using multifaceted interventions and paper reporting systems in reporting PSIs, the implementation of electronic and computer-based reporting systems is gaining momentum. The effective implementation of an electronic-based reporting system should extend into other units beyond critical care units, as it increases the reporting of PSIs, reducing time to make an informed reporting of PSIs and immediate accessibility to information when needed for analysis. The evidence on the implementation of PSI reporting guidelines in SCUs comes from 5 different continents (Asia, Africa, Australia, Europe, and North America). However, the number identified for certain countries within each continent is very minimal.

## Introduction

### Background

Reducing the occurrence of patient safety incidents (PSIs) in the health care system has become a global concern. According to the World Health Organization (WHO) [1], the health care system has still demonstrated unacceptably high rates of PSIs and preventable deaths [1]. Patient Safety remains crucial in the improvement of quality patient care and has been defined by the WHO International Classification for Patient Safety as the reduction of the risk of unnecessary and avoidable harm associated with health care to an acceptable minimum [2,3]. Specialized units (critical care and high care units) are no exception, as critically ill patients tend to be more susceptible and exposed to a complex environment, therefore incurring high rates of preventable PSIs and death [4]. The SCU environment is different from the general wards as it is characterized by highly invasive and complicated procedures that make the patients vulnerable and susceptible to PSIs, leading to prolonged lengths of stay in the hospital. Furthermore, these critically ill patients have comorbidities that are life-threatening, leading to various complications that require immediate interventions. Near misses and PSIs require constant surveillance to improve patient safety in acute and critical care units [5]. Near misses are incidents or situations that have the potential to cause harm but did not reach the patient due to timely intervention, whereas PSI is harm caused by medical mismanagement instead of the underlying disease [6]. PSIs contribute to the cost of care, adding to the burden of the patient and, because of malpractice claims, causing mounting and spiraling costs to the health care system and for society at large [7].

Evidence revealed that in high-income countries, it was estimated that 1 in every 10 patients was harmed while receiving hospital care [8]. This harm might have further increased the length of hospitalization, and use of more health care resources, with cost implications. In low and middle-income countries, 134 million PSIs occurred in hospitals due to unsafe care, which resulted in 2.6 million deaths each year (National Academies of Sciences and Medicine) [9]. A study carried out in the Eastern Mediterranean and Africa revealed that almost one-third of patients who experienced PSIs died, and 4 out of 5 of those incidences were preventable [10]. In South Africa, a study conducted in KwaZulu-Natal revealed that PSIs were still high (47%) and were serious in nature, which might have suggested poor implementation of Patient Safety Incident Reporting and Learning (PSIRL) guidelines and a lack of improvement strategies [11,12]. Therefore, mitigation of the occurrence of PSIs remains an important component in rendering quality patient care and improving clinical outcomes.

Implementation guidelines are critical in guiding the clinical practice and improvement of clinical outcomes. Rosa et al [13] and Weiss [14] affirmed that incorporating evidence into critical care practice is recognized as a crucial requirement for the optimal care of critically ill patients. However, implementation of evidence-based practices is often insufficient due to many barriers, resulting in frequent poor adherence to guideline recommendations in critical care settings [13-16]. In response to mitigate the occurrence of PSIs, a global effort was made by the WHO Member States to develop the implementation intervention strategies relevant to their nations to create a safer environment in the health care system [10]. The World Alliance for Patient Safety first drafted the guidelines for a system to report and learn adverse events, which were updated and revised as WHO Guidelines for PSIRL Systems. In South Africa, it was recommended by the National Department of Health that every health establishment was expected to adhere to the PSRIL system as stipulated in this guideline [1]. A patient safety learning system (sometimes called a critical incident reporting system) refers to structured reporting, collation, and analysis of critical incidents [17]. Nevertheless, failure to reduce the PSI occurrence might be related to the poor implementation of PSIRL guidelines, which might have led to negative clinical outcomes, which made it difficult for the policy makers and health care professionals to handle PSIRL guidelines effectively. There is limited research on evidence that looks at the implementation of PSIRL guidelines in specialized units at a global level.

### Aim and Questions of the Review

The aim of this review is to map the evidence of PSIRL guidelines implemented by health care professionals in specialized care units (SCUs) globally. The broad question of the review is as follows: what evidence exists on the implementation of PSIRL guidelines by health care professionals? What are the gaps in the implementation of PSIRL guidelines in SCUs?

## Methods

### Overview

The Joanna Briggs Institute Scoping Review Methods (2020) and scoping reviews described in the 2020 JBI Manual for Evidence Synthesis [18] were used to map the available evidence on PSIRL guidelines implemented by health care professionals in SCUs. A scoping review protocol was developed and registered with the Open Science Framework. We used the PRISMA-ScR (Preferred Reporting Items for Systematic Reviews and Meta-Analyses extension for Scoping Reviews; Multimedia Appendix 1) checklist: a checklist and explanations guiding the reporting to ensure that the review conforms to the reporting standards of a scoping review [19].

### Eligibility Criteria

The eligibility criteria were guided by the Population, Concept, and Context (PCC) framework, language, timeline, and type of articles, as illustrated in Table 1.

**Table 1:** PCC^a^, language, and timeline to determine the eligibility.

Variable	Inclusion criteria	Exclusion criteria
Population	Heath care workers, health care providers, health care professionals, health personnel, allied health care professionals, nurses, ICU^b^ nurses, intensive care nurses, critical care nurses, and medical doctors.	Health care professionals working in nonspecialized units.
Concept	implementation, practices, intervention strategies, patient safety incident reporting guidelines, voluntary patient safety event reporting, risk management, reporting guidelines, patient safety learning systems, critical incident reporting system, adverse events; errors, critical incident, incident reports, and hospital risk reporting.	Studies on the implementation of patient safety reporting guidelines in medical, surgical, theatre, and emergency departments were excluded.
Context	Specialized or specialized care units, ICUs, critical care units, coronary care units, or renal units, or burns units or high care units, worldwide; globally, African continent, European continent, Asian continent, American continent, Australasian continent, WHO^c^ regions, and United Nations regions.	Studies written in non-English languages and articles on the implementation of PSIRL^d^ in medical, surgical, theatre, and emergency departments were excluded.
Language	Studies written in English were published as of January 2002.	Studies written in non-English languages.
Timeline	From January 2002 to December 2023.	December 2001 and backwards.
Type of studies	Quantitative, qualitative, mixed methods, review articles (systematic, meta-analysis, integrative, and scoping reviews), reports, text and opinion papers, gray literature sources (academic outputs in the form of theses, dissertations, and ongoing research), and professional organizations such as WHO).	Quantitative, qualitative, mixed methods, reviews, reports, text and opinion papers, gray literature sources, and professional organizations with no outcome of interest.

^a^PCC: Population, Concept, and Context.

^b^ICU: intensive care unit.

^c^WHO: World Health Organization.

^d^PSIRL: Patient Safety Incident Reporting and Learning.

### Search Terms

The following search terms and electronic databases were used to identify articles for the scoping review (Table 2).

**Table 2:** Search words.

Criteria	Entries
MeSH^a^ terms	Health care professionals, health plan implementation, hospital risk reporting, globally
Search words	(Health care professionals) OR (Heath care workers or health Care Provider or health personnel) OR (Nurses or ICU nurses or intensive care nurses or critical care nurses) OR (medical doctors) AND Implementation or (practices or Intervention strategies) AND (Patient Safety Incident Reporting guidelines) OR (Voluntary Patient Safety Event Reporting or Risk management or Reporting guidelines or Patient safety learning systems or Critical Incident reporting system or Adverse events or errors or critical incident or incident reports or Hospital risk reporting) AND (Specialised or Specialized care units) OR (ICUs; critical care units or Coronary care unit or Renal units or Burns unit or High care units) AND (Worldwide) or (Globally) or (African continent or European continent or Asian continent or American continent or Australasian continent) OR (WHO regions) OR (United Nations regions)

^a^MeSH: Medical Subject Headings.

### Search Strategy

This is a secondary analysis where the authors screened electronically published health literature from different databases on patient and safety incident reporting electronic systems within the specialized care units in a hospital setting. The researchers followed a 3-step search strategy to find evidence related to the implementation of PSIRL guidelines implemented by health care professionals in SCUs. The reviewers involved the research librarian in designing and refining the search. First, 2 appropriate databases, namely MEDLINE (PubMed or Ovid) and CINAHL, were searched. Thereafter, an analysis of the text words contained in the title and abstract of retrieved papers and the index terms used to describe the studies were followed. A second search was done using all identified keywords and index terms in all the remaining databases, namely PubMed, EBSCO Host, Web of Science, Scopus, African Journals Online, and Sabinet. A search for gray literature was done to locate unpublished evidence, including academic outputs (theses and dissertations) and ongoing research. The authors search gray literature for completed unpublished academic outputs (theses and dissertations) for further evidence on PSI reporting guidelines implemented by the health care professionals in specialized care units. This was accomplished through searching the ProQuest Dissertation and Theses Global, then search engines Google and Google Scholar. Professional organizations such as WHO were also searched. Finally, the reference list of identified articles was searched for additional sources. A sample of a complete search strategy for at least one major database is included in an appendix to the protocol.

### Source of Evidence Selection

The process of source selection was done in 2 stages. The first stage was based on title and abstract examination using the PCC inclusion criteria; thereafter, full-text examination followed as the second stage. All the stages of the review were done by 2 reviewers (TMHG and GC) independently, and any disagreements were solved by consensus. A flowchart of the review process (from the PRISMA-ScR statement) detailing the flow from the search through source selection, duplicates, full-text retrieval, and any additions from the third search, data extraction, and presentation of the evidence was availed. The EndNote (Clarivate) and Ryann (Rayyan Systems, Inc) software were used to manage the search results.

### Data Extraction Process, Presentation, and Analysis

Data extraction and verification were done by 2 reviewers (TMHG & GC). A logical and descriptive summary of the results that align with the specific questions was presented in the form of a charting table. The data included the following key information: authors, year of publication, country where the study was done, populations involved, study methods, guidelines or strategies implemented, and outcomes. Analysis of evidence was done through frequency counts of concepts, population, and context. A narrative summary was done to describe the existing evidence on the implementation of PSIRL guidelines by the health care professionals working in SCUs.

## Results

### Overview

**Figure 1:**
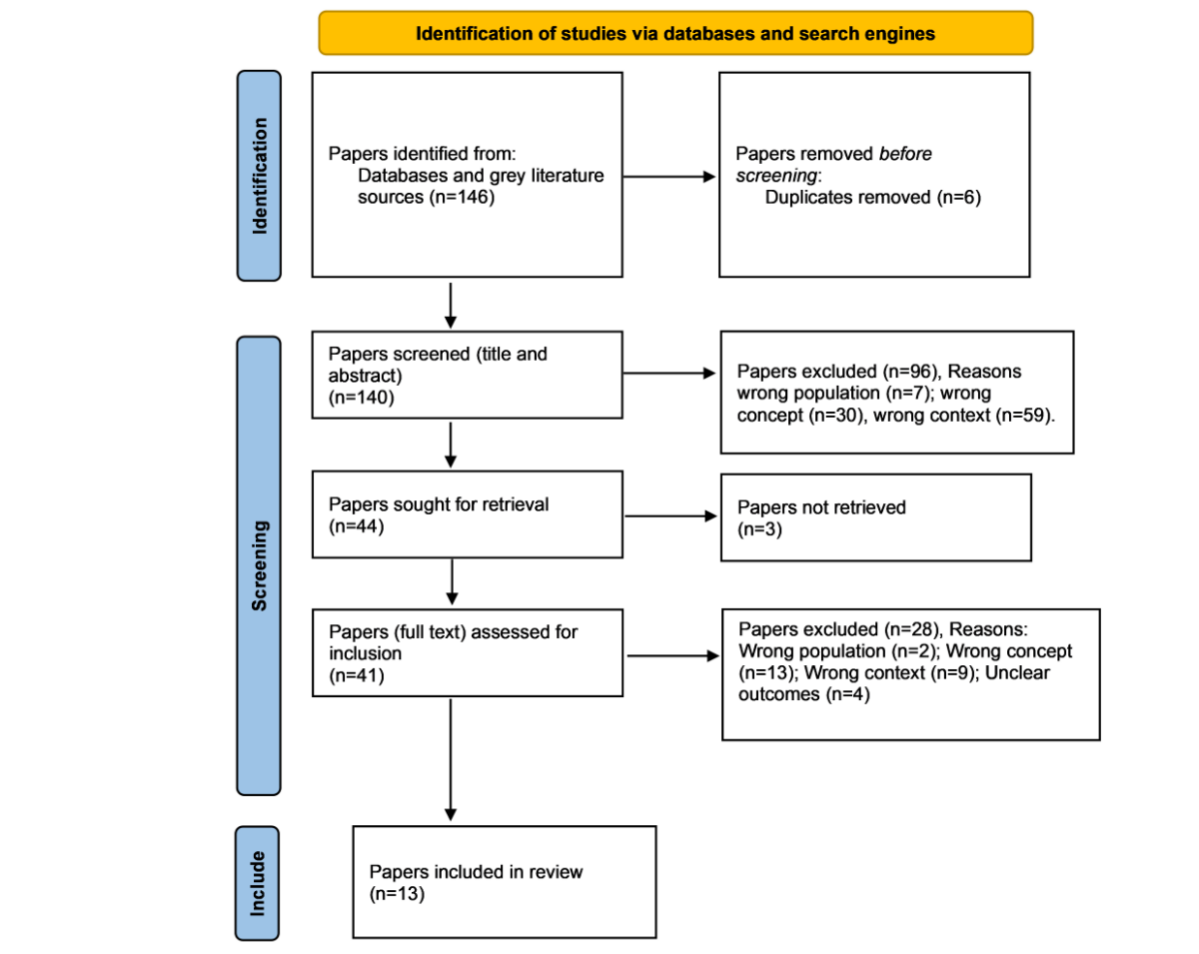
PRISMA (Preferred Reporting Items for Systematic Reviews and Meta-Analyses) flow diagram.

The authors conducted secondary research where the electronically published health literature from different databases on patient and safety incident reporting electronic systems within the SCUs were searched. According to Figure 1, a total of 146 articles were identified from the databases and the search engine Google Scholar. Before the selection process, 6 duplicates were removed to remain with 140 articles for abstract and title screening. After abstract and title screening, 96 articles were excluded based on the following reasons: wrong population (n=7), wrong concept (n=30), and wrong context (n=59). This resulted in the remaining 44 articles for full-text screening. A total of 3 full-text studies could not be retrieved and hence excluded to remain with the 41 full-text studies for second-level screening. Full-text screening yielded the exclusion of 28 articles based on the following reasons: wrong population (n=2), wrong concept (n=13), wrong context (n=9), and unclear outcomes (n=4). As illustrated in Figure 1 below, 13 articles were found to be fitting the inclusion of this scoping review (19-31).

### Publication Trends, Distribution, and Characteristics

The articles used quantitative (n=11) and review (n=2) methodologies, and one was not stated. The 13 selected articles (Table S1 in Multimedia Appendix 2) were published in 2003 (n=1), 2007 (n=1), 2009 (n=1), 2010 (n=1); 2011 (n=1), 2012 (n=1), 2013 (n=1), 2014 (n=1), 2017 (n=2), 2018 (n=1), 2020 (n=1), and 2023 (n=1). The articles were identified from 4 different continents, namely Asia (n=2), Africa (n=2), Australia (n=1), North America (n=1), and Europe (n=8). Most articles come from the Netherlands and Switzerland (both with n=3), followed by South Africa (n=2). Other specific countries like Spain, Ireland, Jordan, Australia, North America, and Japan had only 1 article each.

### Patient Safety Incident Reporting and Learning Guidelines

The nature of implemented patient safety incident reporting and learning guidelines was computer-based (n=11) and paper-based incident reporting (n=2). The reporting system was for all the health care professionals within the SCUs with a focus on patients, staff members, and families. The outcomes of implemented incident reporting and learning guidelines were positive, as evidenced by improved reporting of incidents, including medication errors (n=9). The strategies implemented in these studies included electronic reporting, a voluntary-nonpunitive reporting system, a medication error checklist, an intensive care unit (ICU) incident registry, and a multifaceted intervention that involved the creation of patient safety peer-leadership role, feedback process, interactive dashboards for patient safety data, and education resources accessible through quick response codes, improved the reporting of PSIs. Furthermore, studies that revealed a decreased rate of incidents and errors (n=2) executed various strategies, including clinical information systems, critical patient transport protocols, and handoff communication processes. In addition, 2 studies did not show any change (n=2) in the implementation of the incident reporting and learning guidelines (Table S1 in Multimedia Appendix 2).

## Discussion

### Principal Findings

The review aimed to map the evidence of PSIRL guidelines implemented by health care professionals in SCUs globally. The review was specifically focusing more on the SCUs. However, 4 articles might be nonspecialized ICUs, but they were standardized to be used across the hospital, including the specialized units. The evidence on the implementation of PSIRL guidelines in SCUs comes from 5 different continents (Asia, Africa, Australia, Europe, and North America). However, the number of identified countries within each continent is very minimal. Yet globally, there is a higher percentage of PSIs in SCUs [12]. The implementation of reporting of PSIs in SCUs started to be reported around 2002. However, the frequency of yearly publications remains very low. Meanwhile, health care systems in high-income and transitioning countries have implemented the patient safety reporting system, challenges still exist to reach full scale [20]. As safety culture promoters, incident reporting systems (IRS) serve as a starting point of the learning process to prevent the occurrence of the same incident in the future [21,22].

Implementation of the IRS for risk identification and organizational learning is one way of improving patient safety in health care, including specialized settings [23]. The nature of implemented incident reporting guidelines identified in the review were computer-based reporting [24-31], paper-based incident reporting [32-34], and handoff communication [35] The use of electronic and computer-based reporting system is gaining momentum due to its effectiveness in increasing the reporting of PSI events, reducing time to make an informed reporting of PSIs and immediate accessibility to information when needed for analysis [29,36,37]. Adopting the electronic safety program in specialized units like critical care units will enhance the quality of services that are provided for patients as indicated by Muhsein et al [25].

The paper-based reporting system of PSIs has proved to be inefficient as fewer staff members are willing to report the incidents [38]. However, some SCUs are still using multifaceted paper reporting systems in reporting PSIs, errors related to medication, and critical transportation of patients with positive results [32-34]. On the other hand, Fraenkel et al [26] implemented a computerized clinical information system that replaced paper-based charts of patient observations, clinical records, results reporting, and drug prescribing, which resulted in the reduction of the occurrence of PSIs, less documentation, and more time spent on the patient. According to Ramírez et al [39], the implementation of a hospital IRS, including the systematization of the method and analysis of PSIs by the workshop-trained, results in a reduction in the frequency of PSIs.

In some cases, reporting PSIs and handling them at a unit level (SCUs) is not adequate for developing patient safety, hence the need to use multiple methods to strengthen the overall patient safety culture [23,30]. Griffeth et al [30], involved creation of patient safety peer-leadership role, feedback process, interactive dashboards for patient safety data, and education resources accessible through quick response codes, which resulted in patient safety incident reporting increased by 48%. van der Veer et al [29], used the concept of multiple methods where the ICU incident registry was added to the existing registry, and this resulted in double the number of PSIs reported. In addition, the handoff communication process has proved to lower the number of handoff-related incidents and enhance the satisfaction of nurses [35]. While strong evidence of the current review has revealed the positive outcomes of implementing computer-based reporting systems in SCUs such as critical care units, the systematic review carried out by Frey and Schwappach [31] indicated no improvement in critical incident monitoring after the implementation. Similarly, the use of the multifaceted paper reporting strategy highlighted no change for the PSIs related to airway and indwelling lines [34].

### Strengths

Scoping reviews ensure high-quality articles are included for data extraction. Publications emanating from different countries worldwide depicted comprehensive information on the implementation of the reporting guidelines and strategies, which provided further recommendations. Evidence-based information from the results will be used by policy makers to improve patient safety culture in SCUs.

### Limitations

The review was limited to SCUs only, therefore, pertinent information from other units and other categories of health care professionals may have been disregarded. In addition, only studies published in English were used; therefore, studies published in other languages whose information may have had a valuable contribution were excluded. There is a paucity of literature on the implementation of PSI reporting and learning guidelines worldwide. Therefore, future research studies need to be conducted, especially in Africa.

### Conclusion

The implementation of reporting of PSIs in specialized units started to be reported around 2002; however, the frequency of yearly publications remains very low. Although some specialized units are still using multifaceted interventions and paper reporting systems in reporting PSIs, the implementation of electronic and computer-based reporting systems is gaining momentum. The effective implementation of an electronic-based reporting system should extend into other units beyond critical care units, as it increases the reporting of PSIs, reducing time to make an informed reporting of PSIs and immediate accessibility to information when needed for analysis. The evidence on the implementation of PSI reporting guidelines in SCUs comes from 5 different continents (Asia, Africa, Australia, Europe, and North America). However, the number identified for certain countries within each continent is very minimal.

## Appendix

Multimedia Appendix 1 PRISMA-ScR (Preferred Reporting Items for Systematic Reviews extension for Scoping Reviews) checklist.

Multimedia Appendix 2 Author, year, country, research design, nature of patient safety incident guidelines, type of health care professional, population recipient, the outcome, and recommendations (n=13).

## Abbreviations

ICU: intensive care unit
IRS: incident reporting systems
PCC: Population, Concept, and Context
PRISMA-ScR: Preferred Reporting Items for Systematic Reviews and Meta-Analyses extension for Scoping Reviews
PSIRL: Patient Safety Incident Reporting and Learning
PSI: patient safety incident
SCU: specialized care unit
WHO: World Health Organization

## References

[1] World Health Organization National guideline for patient safety incident reporting and learning in the public health sector of South Africa.

[2] Sherman H, Castro G, Fletcher M, Hatlie M, Hibbert P, Jakob R, Koss R, Lewalle P, Loeb J, Perneger T, Runciman W, Thomson R, Van Der Schaaf T, Virtanen M, World Alliance For Patient Safety Drafting Group, World Alliance for Patient Safety (2009). Towards an international classification for patient safety: the conceptual framework. Int J Qual Health Care.

[3] Runciman W, Hibbert P, Thomson R, Van Der Schaaf T, Sherman H, Lewalle P (2009). Towards an international classification for patient safety: key concepts and terms. Int J Qual Health Care.

[4] Ahmed AH, Thongprayoon C, Schenck LA, Malinchoc M, Konvalinová A, Keegan MT, Gajic O, Pickering BW (2015). Adverse in-hospital events are associated with increased in-hospital mortality and length of stay in patients with or at risk of acute respiratory distress syndrome. Mayo Clin Proc.

[5] Henneman EA, Gawlinski A, Giuliano KK (2012). Surveillance: a strategy for improving patient safety in acute and critical care units. Critical Care Nurse.

[6] da Silva MVO, Caregnato RCA (2019). Intensive care unit: safety and monitoring of adverse events. Journal of Nursing UFPE/Revista de Enfermagem UFPE.

[7] Oyebode F (2013). Clinical errors and medical negligence. Med Princ Pract.

[8] Slawomirski L, Auraaen A, Klazinga NS (2017). The economics of patient safety: strengthening a value-based approach to reducing patient harm at national level.

[9] Giles S, Fletcher M, Baker M, Thomson R Incident reporting and analysis.

[10] World Health Organization (WHO) (2015). Patient Safety Tool Kit.

[11] Gqaleni T, Mkhize S (2023). Healthcare professionals' perception of knowledge and implementation of patient safety incident reporting and learning guidelines in specialised care units, KwaZulu-Natal. South Afr J Crit Care.

[12] Gqaleni TM, Bhengu BR (2020). Analysis of patient safety incident reporting system as an indicator of quality nursing in critical care units in KwaZulu-Natal, South Africa. Health SA.

[13] Rosa RG, Teixeira C, Sjoding M (2020). Novel approaches to facilitate the implementation of guidelines in the ICU. J Crit Care.

[14] Weiss CH (2017). Why do we fail to deliver evidence-based practice in critical care medicine?. Curr Opin Crit Care.

[15] Costa DK, White MR, Ginier E, Manojlovich M, Govindan S, Iwashyna TJ, Sales AE (2017). Identifying barriers to delivering the awakening and breathing coordination, delirium, and early exercise/mobility bundle to minimize adverse outcomes for mechanically ventilated patients: a systematic review. Chest.

[16] Jeffs L, Hayes C, Smith O, Mamdani M, Nisenbaum R, Bell C, McKernan P, Ferris E (2014). The effect of an organizational network for patient safety on safety event reporting. Eval Health Prof.

[17] Health Quality Ontario (2017). Patient safety learning systems: a systematic review and qualitative synthesis. Ont Health Technol Assess Ser.

[18] Peters MD, Marnie C, Tricco AC, Pollock D, Munn Z, Alexander L, McInerney P, Godfrey CM, Khalil H (2020). Updated methodological guidance for the conduct of scoping reviews. JBI Evid Synth.

[19] Tricco AC, Lillie E, Zarin W, O'Brien K, Colquhoun H, Levac D, Moher D, Peters MDJ, Horsley T, Weeks L, Hempel S, Akl EA, Chang C, McGowan J, Stewart L, Hartling L, Aldcroft A, Wilson MG, Garritty C, Lewin S, Godfrey CM, Macdonald MT, Langlois EV, Soares-Weiser K, Moriarty J, Clifford T, Tunçalp Ö, Straus SE (2018). PRISMA extension for scoping reviews (PRISMA-ScR): checklist and explanation. Ann Intern Med.

[20] Koike D, Ito M, Horiguchi A, Yatsuya H, Ota A (2022). Implementation strategies for the patient safety reporting system using consolidated framework for implementation research: a retrospective mixed-method analysis. BMC Health Serv Res.

[21] Torre-Pérez LDL, Granés L, Prat Marín A, Bertran M (2023). A hospital incident reporting system (2016-2019): learning from notifier's perception on incidents' risk, severity and frequency of adverse events. J Healthc Qual Res.

[22] Harsul W, Irwan AM, Sjattar EL (2020). The relationship between nurse self-efficacy and the culture of patient safety incident reporting in a district general hospital, Indonesia. Clinical Epidemiology and Global Health.

[23] Sahlström M, Partanen P, Turunen H (2018). Patient-reported experiences of patient safety incidents need to be utilized more systematically in promoting safe care. Int J Qual Health Care.

[24] Kabane S (2013). An Evaluation of the Effectiveness of a Hospital Clinical Adverse Event Prevention Programme.

[25] Muhsein AS, Al-Slehat MA, Ghurra MFB, Al-Shnaaq AAA, Salman ZS, Saleh AM (2017). The impact of implementing electronic safety program on patient safety in intensive care unit, Istishari Hospital. JMENAS.

[26] Fraenkel DJ, Cowie M, Daley P (2003). Quality benefits of an intensive care clinical information system. Crit Care Med.

[27] Snijders C, Kollen BJ, van Lingen RA, Fetter WPF, Molendijk H, NEOSAFE Study Group (2009). Which aspects of safety culture predict incident reporting behavior in neonatal intensive care units? A multilevel analysis. Crit Care Med.

[28] Brunsveld-Reinders AH, Arbous MS, De Vos Rien, De Jonge Evert (2016). Incident and error reporting systems in intensive care: a systematic review of the literature. Int J Qual Health Care.

[29] van der Veer S, Cornet R, de Jonge E (2007). Design and implementation of an ICU incident registry. Int J Med Inform.

[30] Griffeth EM, Gajic O, Schueler N, Todd A, Ramar K (2023). Multifaceted intervention to improve patient safety incident reporting in intensive care units. Journal of patient safety.

[31] Frey B, Schwappach D (2010). Critical incident monitoring in paediatric and adult critical care: from reporting to improved patient outcomes?. Curr Opin Crit Care.

[32] Truter A, Schellack N, Meyer JC (2017). Identifying medication errors in the neonatal intensive care unit and paediatric wards using a medication error checklist at a tertiary academic hospital in Gauteng, South Africa. SAJCH.

[33] García PN, Avión RC, Ruiloba ML, Pérez JR, Dobarro AB, García AR (2020). Retrospective study of security in the transfer of critical patients after application of methodology for risk management. Revista Española de Anestesiología y Reanimación (English Edition).

[34] Pagnamenta A, Rabito G, Arosio A, Perren A, Malacrida R, Barazzoni F, Domenighetti G (2012). Adverse event reporting in adult intensive care units and the impact of a multifaceted intervention on drug-related adverse events. Ann Intensive Care.

[35] Ibrahim MA (2014). Improving nursing handoff process in the cardiovascular intensive care unit.

[36] Kanda H (2011). Development of an online incident-reporting system for management of medical risks at hospital. Yakugaku Zasshi.

[37] Giles S, Fletcher M, Baker M, Thomson R (2005). Incident reporting and analysis. Patient Safety: Research into Practice.

[38] Gao X, Yan S, Wu W, Zhang R, Lu Y, Xiao S (2019). Implications from China patient safety incidents reporting system. Therapeutics and clinical risk management.

[39] Ramírez E, Martín A, Villán Y, Lorente M, Ojeda J, Moro M, Vara C, Avenza M, Domingo MJ, Alonso P, Asensio MJ, Blázquez JA, Hernández R, Frías J, Frank A, SINOIRES Working Group (2018). Effectiveness and limitations of an incident-reporting system analyzed by local clinical safety leaders in a tertiary hospital: Prospective evaluation through real-time observations of patient safety incidents. Medicine (Baltimore).

