# Supplementary material for: Patient Safety Incident Reporting and Learning Guidelines Implemented by Health Care Professionals in Specialized Care Units: Scoping Review
Source: J Med Internet Res. 2024 Oct 4;26:e48580. doi: 10.2196/48580 (PMC11489802; doi:10.2196/48580)
Supplement: Multimedia Appendix 2 [file jmir_v26i1e48580_app2.docx]

**Table S1. Author, year, country, research design, nature of PSI guidelines, type of health care professional, population recipient, the outcome, and recommendations (n=13).**

| **Author, year, country** | **Research design** | **Patient safety incident reporting guideline** | | | | **Recommendation** |
| --- | --- | --- | --- | --- | --- | --- |
|  |  | **Nature of PSI guidelines or strategy** | **Type of health care professional** | **Population recipient** | **The outcome of the implementation** |  |
| **African continent** | | | | | | |
| Kabane S [1], 2013; South Africa | Interventional study | Computerized incident reporting system; Hospital | Health care workers | Patients | Increased reporting of incidents is significantly higher in the intervention compared with the control sites. The findings reported for improving the safety culture were largely positive, but only in selected areas. The researcher and the management of the Free State Department of Health are convinced that this model has reduced patient safety risks at its hospitals indeed. | The use of the patient safety risk reduction model should serve as a fundamental framework for enhancing patient safety and improving health care quality. This model can be effectively used by any province or country that is delivering health care in a setting with limited resources, with some necessary modifications. |
| Truter et al [2], 2017; South Africa | Prospective, quantitative design with a descriptive approach. | Medication error checklist | NICU staff | Pediatric patients and staff | Higher incidences of medication errors were reported. | It is recommended to implement a formal system for documenting these errors, in addition to regular talks among the interdisciplinary team about preventive measures. |
| **Asian Continent** | | | | | | |
| Kanda H [3], 2011; Japan | Not clearly stated; Hospital | An online report input system | All health care professionals | Patients | 307 and 789 cases were reported within 24 h and 48 h, respectively, indicating that the first report was input mostly without delay by the operational guidelines. Cases that took more than 2 weeks to be reported would likely have gone unreported had there not been a first report to indicate and confirm that an incident had even occurred. | It is necessary to include specific information about occurrences in this system using unrestricted text, which provides details that cannot be obtained using multiple-choice options like in typical reporting systems. |
| Muhsein et al [4], 2017; Jordan | Health Services Executive change module | Electronic safety program; ICU | ICU nursing staff | Adult patients and staff | Implementing an electronic safety program within Health Services Executive change module would protect patient safety and help health care providers to be aware of patients’ conditions and quality of care. | It is crucial to use electronic safety measures in health care centers and hospitals to enhance the quality of patient service. |
| **Australia Continent** | | | | | | |
| Fraenkel et al [5], 2003; Australia | Longitudinal observational study; adult general intensive care unit | Clinical information system to replace paper-based charts of patient observations, clinical records, results reporting, and drug prescribing | Nursing staff | Patients | A significant reduction in the rates of medication, intravenous therapy, and ventilator incidents. There was a trend toward a reduction in pressure sores. A positive perception of the clinical information system by nursing staff, with less time spent on documentation and more time in patient care. Nursing staff recruitment and retention improved after the clinical information system implementation. | The introduction of a comprehensive clinical information system resulted in significant enhancements in important quality measures, favorable perceptions among nursing staff, and certain positive effects on resources. |
| **European continent** | | | | | | |
| Garcia, et al [6], 2020; Spain | Retrospective descriptive analysis; Intensive care unit | Critical patient transport protocol and its application through checklists | ICU Doctors, nurses, hospital Managers, representatives from the quality unit | ICU patients | The rate of safety-related incidents was less. Over time, adherence to protocol compliance increased. | Implementing a critical patient transport policy and using checklists can effectively decrease the occurrence of adverse events and nondamaging mishaps in these patients. |
| Snijders, et al [7], 2009; Netherlands | Descriptive survey; NICUs and one pediatric surgical ICU. | a specialty-based, voluntary, nonpunitive incident reporting system | All ICU personnel | Patients | The number of self-reported incidents increased after the intervention. It was positively associated with a nonpunitive response to error and negatively associated with overall perceptions of safety and hospital management support for patient safety. | The absence of punishment for mistakes, the support from hospital management for patient safety, and the general sense of safety are factors that determine the likelihood of incident reporting in the NICU. |
| Brunsveld-Reinders et al [8], 2016; Netherlands | Systematic review; Adult intensive care | ICU incident reporting systems | All ICU health care professionals | ICU patients | All the IRSs still need to fulfill the WHO checklist criteria. This resulted in an administrative report system rather than the much-desired instrument for practice change. Increase in quality as an IRS can only effectively contribute to improving patient safety and quality of care if more attention is given to analyzing incidents and feedback. | Health care organizations should prioritize the recruitment of skilled professionals who can effectively provide feedback on information and improvement measures, as well as assist in implementing and monitoring the impact of these efforts. |
| Pagnamenta et al [9], 2012 Southern Switzerland | Before-and after-study design. Self-reporting questionnaire and Risk index scores | Multifaceted paper reporting strategy | Health caregivers | Adult ICU patients | mean risk-index score for medication errors improved, as well as communication. No change for the airway and indwelling lines related to AEs | Effective implementation of any risk assessment system requires educational interventions, protocol execution, thorough training, and the use of relevant examples to ensure maximum consistency in scoring. |
| Frey and Schwappach [10], 2010; Switzerland | Review; Paediatric and adult critical care | Risk scoring of critical incident reports and root cause analysis | Pediatric and adult critical care staff | Patients and their families | Hard outcome criteria have shown no improvement in critical incident monitoring, e.g., standardized mortality ratio. | Integrating system modifications based on event reports and incorporating the knowledge and insights of patients and their families can enhance patient outcomes. |
| Van der Veer et al [11] 2007; Netherlands | Implementation design | ICU Incident registry added to the existing registry | Nurses and physicians | ICU patients | The number of reported events was more than doubled | Further refinement and modifications are necessary. |
| Muhammed, 2014 (Dissertation) [12]; Ireland | Survey on Patient safety culture | Handoff communication process | ICU nurses | ICU patients | The project evaluation results showed a decline in the percentages of handoff-related incidents and improved the nurses’ satisfaction. | Similar improvement projects are expected to be conducted. |
| **North America Continent** | | | | | | |
| Griffeth et al [13], 2023; USA | a multifaceted intervention developed using a quality improvement methodology | The study intervention involved the creation of a patient safety peer-leadership role, a feedback process, interactive dashboards for patient safety data, and educational resources that were accessible via quick response codes. | ICU staff | Patients | This multifaceted quality improvement intervention increased patient safety incident reporting in the ICUs. Intensive care unit patient safety incident reporting increased by 48% after intervention. Near misses were the most common incident report. | An increase in ratings of learning culture and support for staff underlines the importance of a well-functioning patient safety incident reporting system. |

**References**

1. *Kabane S. An Evaluation of the Effectiveness of a Hospital Clinical Adverse Event Prevention Programme. Pretoria, South Africa: University of Pretoria; 2013.*
2. *Truter A, Schellack N, Meyer JC. Identifying medication errors in the neonatal intensive care unit and paediatric wards using a medication error checklist at a tertiary academic hospital in Gauteng, South Africa. SAJCH 2017; 11(1):5-10.*
3. *Kanda H. Development of an online incident-reporting system for management of medical risks at hospital. Yakugaku Zasshi 2011; 131(9):1353-1359.*
4. *Muhsein AS, Al-Slehat MA, Ghurra MFB, Al-Shnaaq AAA, Salman ZS, Saleh AM. The impact of implementing electronic safety program on patient safety in intensive care unit, istishari hospital. JMENAS 2017; 3(2):35-40.*
5. *Fraenkel DJ, Cowie M, Daley P. Quality benefits of an intensive care clinical information system. Crit Care Med 2003; 31(1):120-125.*
6. *García PN, Avión RC, Ruiloba ML, Pérez JR, Dobarro AB, García AR. Retrospective study of security in the transfer of critical patients after application of methodology for risk management. Revista Española de Anestesiología y Reanimación (English Edition) 2020; 67(3):119-129.*
7. *Snijders C, Kollen BJ, van Lingen RA, Fetter WPF, Molendijk H, NEOSAFE Study Group. Which aspects of safety culture predict incident reporting behavior in neonatal intensive care units? A multilevel analysis. Crit Care Med 2009; 37(1):61-67.*
8. *Brunsveld-Reinders AH, Arbous MS, De Vos Rien, De Jonge Evert. Incident and error reporting systems in intensive care: a systematic review of the literature. Int J Qual Health Care 2016; 28(1):2-13.*
9. *Pagnamenta A, Rabito G, Arosio A, Perren A, Malacrida R, Barazzoni F, Domenighetti G. Adverse event reporting in adult intensive care units and the impact of a multifaceted intervention on drug-related adverse events. Ann Intensive Care 2012; 2(1):47.*
10. *Frey B, Schwappach D. Critical incident monitoring in paediatric and adult critical care: from reporting to improved patient outcomes? Curr Opin Crit Care 2010; 16(6):649-653.*
11. *van der Veer S, Cornet R, de Jonge E. Design and implementation of an ICU incident registry. Int J Med Inform 2007; 76(2-3):103-108.*
12. *Ibrahim MA. Improving nursing handoff process in the cardiovascular intensive care unit. Royal College of Surgeons in Ireland; 2014. [accessed 2024-09-05].*
13. *Griffeth EM, Gajic O, Schueler N, Todd A, Ramar K. Multifaceted intervention to improve patient safety incident reporting in intensive care units. Journal of patient safety 2023; 19(7):422-428.*
